# The Financial Report of the King's Fund

**Published:** 1912-09-14

**Authors:** 


### The Hospital

The Workers' Newspaper of Administrative Medicine and Institutional Life, Administration, National Insurance and Health.

No. 1367, Vol. LII. SATURDAY, SEPTEMBER 14, 1912. PRICE ONE PENNY.

### THE FINANCIAL REPORT OF THE KING'S FUND.

The Ninth Annual Report on the ordinary expenditure of one hundred and seven London hospitals for the year 1911 has just been issued by the Secretaries of the King's Fund. The main point noted in the Report is the increased cost per bed and per attendance of out-patients, which, in combination, amounts to an extra sum of nearly £10,000. Against this, as the Secretaries point out, must be set the amount of £47,000 achieved in economies by the hospitals since the first Report was issued eight years ago. Mr. Maynard has analysed the tables contained in the Report with great ability and clearness: his introduction is concise and to the point, and should be read by everyone who is interested in hospital finance. Yet even more interesting is a comparison of the tables themselves, such as every reader may make for himself. Such a comparison shows clearly the enormous divergences between various hospitals. Some of these discrepancies are easily accounted for. Special hospitals necessarily cost more in upkeep than general hospitals where the cost is distributed over a large number of beds. Small hospitals, again, are relatively more expensive than large ones. Here and there individual peculiarities considerably augment the annual expenditure. But even when all allowances have been made for these wide differences there are a few points which so forcibly strike the reader's imagination that they at once raise the question whether or not a still further economy may be effected by co-operation between the various institutions.

Let us take, for instance, in this connection one set of tables, those dealing with laundry expenses. We see that after excluding such special institutions as Mount Vernon Hospital, the London Fever, and the Lock Hospital (in all of which special disinfecting plant is used in the laundry, with consequent increase of expenses), the total cost of washing a dozen articles varies from four to twelve pence (excluding fractions of a penny) when the work is done on the hospital premises; from five to twenty-six pence when the work is done outside; and from two to twelve pence when it is done partly on and partly off the premises. The enormous differences shown by these figures can scarcely be explained in any other way than by postulating a certain amount of extravagance in some institutions. We do not mean to imply that such extravagance is wilful: in many cases, we are confident, it is due to factors which are hardly recognised by the authorities. One of these undoubtedly is the use of hard water, for, as we have frequently pointed out, the employment of decalcified water in the laundry saves the institution an appreciable sum annually. A comparison of these laundry tables with the figures for Brussels, where a central hospital laundry is available, clearly proves that a further saving may be effected by co-operation. It should not be impossible, nor yet very difficult, for the hospitals in certain districts to combine and erect their common laundry plant with a central system for removing and collecting soiled linen. Such a system of centralisation will create a new department, of course, but it will have compensating advantages, chief of which would be the saving of money now needlessly spent on laundry work. Similar co-operative work in the drug department may possibly effect a similar economy, although we confess that central dispensaries do not appeal to us so strongly as does a central laundry. But when we find on page 102 of the Report that there are huge variations in the prices paid by different institutions for the same materials we are inclined to believe that a central buying department would be a paying innovation for our metropolitan institutions. That the highest price paid for bismuth carbonate was 12s. 6d. per pound and the lowest 3s., may be explained by two suppositions: first, that the low price was paid at a time of year when the drug was extraordinarily cheap; and, secondly, that the high price was charged when the price had risen, and to a hospital that only bought a relatively small quantity. But a similar supposition will not explain the discrepancy between 18s. and 7s., the highest and lowest prices per dozen for half-minute clinical thermometers, for there is relatively little fluctuation in the price of these articles. The only feasible explanation for these and similar discrepancies is that the low-priced drugs and articles are purchased in gross by tender, and that in this way the cheapest, though not necessarily the best, material is obtained. Such a method undoubtedly has its advantages, for the quality of the goods can be ensured by personal inspection on the part of the secretary or superintendent. But low-priced goods obtained through tender in one hospital and high-priced goods obtained by purchase in shops at another hospital mean inequality of material, and it is desirable that there should be a certain measure of uniformity in the various hospitals. Without such uniformity future statistics of treatment will be largely vitiated; or at least their proper interpretation will be most difficult. Such uniformity, we venture to think, can be secured, in a measure at least, by the establishment of a central buying station which would cater for all the hospitals in its district. The question has been discussed by hospital authorities in recent times, but in view of the statistics contained in the King's Fund Reports it is worth while to deal with it once more.

For the present we commend Mr. Maynard's comments and the tables on which they are based to the attention of all hospital secretaries, whether metropolitan or provincial.

